# Mechanism of Ulcerative Colitis-Aggravated Liver Fibrosis: The Activation of Hepatic Stellate Cells and TLR4 Signaling Through Gut-Liver Axis

**DOI:** 10.3389/fphys.2021.695019

**Published:** 2021-09-17

**Authors:** Yu-Feng Liu, Guo-Chao Niu, Chen-Yang Li, Jin-Bo Guo, Jia Song, Hui Li, Xiao-Lan Zhang

**Affiliations:** ^1^Department of Gastroenterology, The Second Hospital of Hebei Medical University, Shijiazhuang, China; ^2^Department of Gastroenterology, Dingzhou People’s Hospital of Hebei Province, Dingzhou, China

**Keywords:** liver fibrosis, ulcerative colitis, intestinal homeostasis, hepatic stellate cells, intestinal tight junction, TLR4 signaling, gut-liver axis

## Abstract

**Background:** The progression of liver disorders is frequently associated with inflammatory bowel disease through the gut-liver axis. However, no direct evidence showed the mechanisms of ulcerative colitis (UC) in the development of liver fibrosis *per se*. Thus, this study aimed to evaluate the effects of UC on liver fibrosis and its potential mechanism in the experimental model.

**Methods:** Male C57BL/6 mice were allocated into five groups (*n* = 10 per group) to receive either drinking water (control), 2% dextran sulfate sodium (DSS), olive oil, carbon tetrachloride (CCl_4_) or DSS + CCl_4_ for 4 cycles. Blood was collected for biochemical analysis. Colons were excised for the evaluation of colon length and morphological score. Liver, colon, and mesenteric lymph nodes (MLNs) were collected for histopathological staining, expression analysis, and bacterial translocation assay to evaluate the inflammation, fibrosis, the activation of hepatic stellate cells (HSCs), and gut barrier function.

**Results:** DSS caused severe colitis in mice treated or treated with CCl_4_, as evident from the elevation of disease activity index (DAI), histological abnormalities, and increased pro-inflammatory cytokines (TNF-α, IFN-γ, and IL-17A). Histopathological staining revealed that DSS treatment aggravated the CCl_4_-induced extracellular matrix deposition, liver fibrosis, and inflammation in mice. Additionally, biochemical and expression analysis indicated the DSS treatment caused the increase of hydroxyproline and pro-inflammatory cytokines, as well as the abnormal liver function indexes in CCl_4_-induced mice. Gut barrier function was impaired in DSS- and DSS + CCl_4_-treated mice, manifesting as the increase in bacterial translocation and lipopolysaccharide level, and the reduction in tight junction proteins (occluding, claudin-1 and ZO-1) expression. Further, the activations of HSCs and TLR4 signaling pathway were observed after DSS + CCl_4_ treatment, presenting with the increase in expression of α-SMA, vimentin, TGF-β, collagen type I, collagen type II, TIMP-2, TLR4, TRAF6, and NF-κB p65, and a decrease in GFAP and MMP-2 expression.

**Conclusion:** The present study verified that UC aggravated CCl_4_-induced liver injury, inflammation, and fibrosis in mice through the gut-liver axis. Gut barrier dysfunction in UC leads to bacterial translocation and elevated lipopolysaccharide, which may promote the activation of TLR4 signaling and HSCs in the liver.

## Introduction

Liver fibrosis is a common disease associated with various liver disorders such as viral hepatitis, and alcoholic liver, which often leads to gradual loss of liver function (Shiha et al., 2017). It has the potential to progress into cirrhosis, even liver cancer, and liver failure if not necessarily prevented (Aydın and Akçalı, 2018). Moreover, the progression of fibrosis, and particularly cirrhosis, is responsible for significant morbidity and mortality (Berumen et al., 2021). However, liver transplantation is still the only available curative treatment for patients with end-stage of liver fibrosis at present (Koyama et al., 2016). In recent years, liver fibrosis is considered a dynamic and potentially bidirectional process that has an inherent capacity for recovery and remodeling (Hintermann and Christen, 2019), which translates new possibilities into the development of anti-fibrotic therapies. Despite numerous therapeutic targets have been identified in liver fibrosis, unfortunately, no clinical trial has so far provided unequivocal evidence for a complete reversal of cirrhosis (Povero et al., 2010; Pellicoro et al., 2014). Accordingly, identifying the critical pathways and mechanisms in liver fibrosis is likely to provide novel insights for clinical translation.

Notably, a considerable number of studies have been indicated that the progression of liver disorders is frequently associated with inflammatory bowel disease through the gut-liver axis (Frasinariu et al., 2013), as the blood supply in the liver mostly comes from the intestine through the portal vein (Seki and Schnabl, 2012). Particularly, liver disorders are typical extra-intestinal manifestations in ulcerative colitis (UC). Several previous studies have demonstrated the dextran sodium sulfate (DSS)-induced colitis promotes liver damage, inflammation, fibrogenesis, and even tumorigenesis in the non-alcoholic steatohepatitis (NASH) model (Gäbele et al., 2011; Trivedi and Jena, 2013; Achiwa et al., 2016). Remarkably, little attention was paid to liver fibrosis *per se* for many years, even though almost all morbidity of liver diseases can be tied directly to progressive fibrosis (Albanis and Friedman, 2001). Currently, no direct evidence has shown the mechanisms of UC in the development of liver fibrosis *per se*. Therefore, elucidating the role of DSS-induced colitis in the liver fibrosis model would provide additional evidence for the development of anti-fibrotic therapies.

Gut-liver axis is widely recognized to be implicated in the pathogenesis of liver disorders and is emerging as the focus of clinical research increasingly (Wiest et al., 2017). Early studies identified the increased level of portal lipopolysaccharide (LPS) and intestinal permeability in experimental NASH or alcoholic liver disease (ALD), suggesting the gut barrier dysfunction (Szabo, 2015). More recently, evidence from clinical patients proposed a new viewpoint on the role of the gut-liver axis in the pathogenesis of liver disorders (Haderer et al., 2021). Liver cirrhosis could result in a reduction in the thickness of the colonic mucus layer, which allows bacteria-to-epithelial cell contact, and subsequently bacteria led to a marked reduction of cell-to-cell junctions (Haderer et al., 2021). Furthermore, the destruction of intestinal tight junctions increases intestinal permeability, leading to the transport of intestinal bacterial products to the liver via the portal vein (Matsuda and Seki, 2020). These bacterial products stimulate innate immune receptors, namely Toll-like receptors (TLRs), eventually activating downstream pathways involved in liver inflammation and fibrogenesis (Frasinariu et al., 2013; Hu et al., 2020). However, the evidence to support this pathogenesis mechanism of LPS-activated TLR4 signaling comes largely from experimental NASH, data from carbon tetrachloride (CCl_4_)-induced liver fibrosis model has not been reported currently. As reported, the development of different liver fibrosis (such as hepatotoxic and cholestatic) depends on the etiology of the underlying liver disease (Kisseleva and Brenner, 2021). Thus, the purpose of this study was to evaluate the effects of DSS-induced colitis on liver inflammation and fibrosis in mice with CCl_4_-induced liver fibrosis, hoping to provide additional and direct evidence that highlight the mechanisms of gut–liver axis relevant to liver fibrosis.

## Materials and Methods

### Animals and Experimental Protocol

Male C57BL/6 mice (weight 18–22 g; age 6–8 weeks) were obtained from Laboratory Animal Centre of Hebei Medical University (Shijiazhuang, Hebei, China). All mice were maintained under standard conditions (temperature, 23 ± 2°C; humidity, 70–75%; lighting regimen, 12-h light/dark cycle), and free access to food and water. All experimental protocols were conducted according to the Institutional Animal Care guidelines and approved by the Laboratory Animal Ethics Committee of Hebei Medical University.

Fifty mice were randomly allocated into five experimental groups: control, DSS, olive oil, CCl_4_, and DSS + CCl_4_ (*n* = 10 per group) and received the assigned treatments. Mice in the control group received normal drinking water throughout the study. Mice in the DSS group received 2% w/v DSS (40000–50000 MW, dissolved in drinking water, Sigma, United States) for 4 cycles (on day 1–5, 8–12, 15–19, and 22–26, respectively) and distilled water (remission period between each cycle) to induce the chronic colitis. Mice in the olive oil group received normal drinking water and intraperitoneal injection of sterile olive oil twice a week and served as a control for CCl_4_ group. Mice in the CCl_4_ group were given normal drinking water and the intraperitoneal injection of 5% CCl_4_ dissolved in sterile olive oil (10 μl/g body weight). CCl_4_ + DSS group received 4-cycle administration of 2% DSS and intraperitoneal injection of CCl_4_. DSS was applied in cycles and each cycle consisted of 5 days DSS administration followed by a 2-day interval with normal drinking water. CCl_4_ was injected twice a week. The cycles were repeated throughout the experimental period of 4 weeks. During the experimental period, the clinical activity of colitis was blindly assessed by an independent observer to determine disease activity index (DAI), including weight loss, diarrhea, and bloody stools and recorded. DAI was calculated using DAI scoring system described by Barrett et al. (2012). Colon myeloperoxidase (MPO) activity was also measured to assess the colonic injury and inflammation using the Myeloperoxidase Activity Assay Kit (Jiancheng Bioengineering Institute, Nanjing, China) according to the manufacturer’s protocol.

### Sample Preparation

Mice were anesthetized with pentobarbital and sacrificed by cervical dislocation. Blood was collected from arteria femoralis of mice at the time of sacrificed after a 12-h fast. Serum samples were separated from blood by the centrifugation at 3000 rpm for 15 min and stored at –80°C until the biochemical analysis. Colons were excised and dried after washing in phosphate buffer-saline (PBS), then used for the evaluation of colon length and morphological score, as previously described (Zhang et al., 2020). Liver was also resected after sacrificed. Part of liver or colon was fixed in 4% paraformaldehyde, embedded into paraffin, and cut to 4 μm sections for the histological analysis. Remaining liver or colon tissues were stored for the subsequent analysis, such as detection of hydroxyproline in liver and expression analysis of protein.

### Biochemical Analysis

Serum lipopolysaccharide (LPS), alanine aminotransferase (ALT), glutamic oxalacetic transaminase (AST), albumin (ALB), total bilirubin (TBIL), direct bilirubin (DBIL), and hydroxyproline were measured using commercially available kits (Jiancheng Bioengineering Institute, Nanjing, China). The analyses were performed in BECKMAN COULTER CX9Automatic Aralyzer (BECKMAN, United States) or UV-2000 spectrophotometer (Unicosh, Shanghai, China).

### Histopathological Staining

Paraffin-embedded colon and liver sections were, respectively, stained with hematoxylin and eosin (H&E) for the histological evaluation of inflammation, extent, gland damage, and cell infiltration. Liver sections were stained with Masson’s trichrome (MT) or Sirius red for fibrosis evaluation. The stained sections were examined under a microscope (Olympus BX51, Japan). Histological score was evaluated according to the established scale based on inflammatory and epithelial parameters (Aranda et al., 1997). Fibrotic stage was graded on a 0–4 scale, including no fibrosis, portal, periportal, or bridging fibrosis and cirrhosis, respectively. Fibrotic area were quantified after Sirius red staining.

### Bacterial Translocation Assay

After mice were sacrificed, mesenteric lymph nodes (MLN) were resected under aseptic conditions. MLN samples were washed in 200 μl PBS containing gentamicin (50 μg/mL) and homogenized in ice-cold PBS. Tissue homogenates were seeded in Luria-Bertani plate at 37°C for 24 h. The number of colonies was counted based on the dilution ratio and expressed as colony forming unit (CFU). Bacterial translocation were assessed by the amount of bacteria per gram (CFU/g).

### Immunohistochemistry (IHC) Staining

IHC staining was conducted according to standard protocol. The paraffin-embedded liver or colon tissues were sequentially deparaffinized, rehydrated and washed. Sections were then treated with pH 6.0 sodium citrate buffer, followed by blocking with 3% H_2_O_2_ and goat serum. Next, the following primary antibodies: anti-claudin-1 (Santa Cruz, United States), anti-occludin (Santa Cruz, United States), anti-ZO-1 (Abcam United States), anti-tumor necrosis factor-α (TNF-α, Bio Legend, United States), anti-interferon-γ (IFN-γ, Bio Legend, United States), anti-interleukin-17A (IL-17A, Santa Cruz, United States), anti-TLR4 (Santa Cruz, United States), anti-TNF receptor-associated factor 6 (TRAF6, Santa Cruz, United States), anti-nuclear factor-κB p65 (NF-κB p65, Santa Cruz, United States), anti-α-smooth muscle actin (α-SMA, Abcam, United States), anti-transforming growth factor-β (TGF-β, Santa Cruz, United States), anti-Collagen type I (Col I, Santa Cruz, United States), anti-Collagen type III (Col III, Santa Cruz, United States), anti-matrix metalloproteinase-2 (MMP-2, Proteintech, United States), anti-tissue inhibitor of MMP-2 (TIMP-2, Santa Cruz, United States), and anti-glyceraldehyde-3-phosphate dehydrogenase (GAPDH, Santa Cruz, United States) were used to stain the target proteins. Peroxidase-conjugated antibody were used as secondary antibody. Detection was performed using diaminobezidin (Dako, Denmark) as chromogen. Images were acquired under fluorescent microscopy (Olympus, Japan) and analyzed using Image-Pro Plus 6.0 software (Media Cybernetics Inc., United States).

### Quantitative Real-Time Polymerase Chain Reaction (qRT-PCR) Analysis

Total RNA was prepared from the liver or colon tissues using Trizol reagent (Invitrogen, Carlsbad, CA, United States) according to the manufacturer’s instructions. Then, cDNA was synthesized from total RNA by iScript select cDNA synthesis kit (Bio-Rad, Hercules, CA, United States). Quantitative real-time PCR was performed with iTaq Universal SYBR Green Supermix (Bio-Rad) by CFX96 Touch real-time PCR detection system (Bio-Rad, Hercules, CA, United States). GAPDH was used as the endogenous control. The relative quantities were normalized to endogenous control values and calculated by using the 2^–△△Ct^ method. Sequences of primers are described in Table 1.

**TABLE 1:** Primer sequence for qRT-PCR.

Gene	Forward primer (5′–3′)	Reverse primer (5′–3′)
Claudin-1	TCCTTGCTGAATCTGAACA	AGCCATCCACATCTTCTG
Occludin	GAGGAGAGTGAAGAGTACAT GGGCTG	GTCTGTCATAATCTCCCACC ATCCT
ZO-1	TCATCCCAAATAAGAACAGAGC	GAAGAACAACCCTTTCATAAGC
TNF-α	GGAAAGGACGGACTGGTGTA	TGCCACTGGTCTGTAATCCA
IFN-γ	GGCAAGTTCAACGGCACAG	CGCCAGTAGACTCCACGACAT
IL-17A	GGAAAGGACGGACTGGTGTA	TGCCACTGGTCT GTAATCCA
TLR4	TTTATTCAGAGCCGTTGG	CCCATTCCAGGTAGGTGT
TRAF6	GTATCCGCATTGAGAAGC	GCAGTGAACCATCCGTGT
NF-κB p65	AAGGATTCGAGCAGTTAG	AAGAGTTGGTGATAGGCT
GFAP	CTGGAGGTTGAGAGGGACAA	CTGGAGGTTGAGAGGGACAA
Vimentin	CGAAAACACCCTGCAATCTT	CGAAAACACCCTGCAATCTT
α-SMA	TGCTGTCCCTCTATGCCTCT	GAAGGAATAGCCACGTCAG
TGF-β	AACTAAGGCTCGCCAGTCC	GCGGTCCACCATTAGCAC
Collagen type I	GCTGGAAAGGAAGGGATT	GGGAGCACCAAGAAGACC
Collagen type III	CCCACAGCCTTCTACACCT	CCAGGGTCACCATTTCTC
MMP-2	GGAATGCCATCCCTGATAACCT	TCCAAACTTCACGCTCT TGAGAC
TIMP-2	GAAGGAGTATCTAATTGCAG GAAAGG	TCTGGGTGATGCTAAGCGTGTC
GAPDH	GGCAAGTTCAACGGCACAG	CGCCAGTAGACTCCACGACAT

### Western Blot Analysis

Samples of liver or colon were homogenized in lysis buffer (Beyotime, Beijing, China) and centrifuged. Protein concentration of the supernatant was determined with coomassie brilliant blue assay. The extracts containing equal quantities of proteins (80 μg) were subjected to 10% polyacrylamide gel electrophoresis and then transferred to a polyvinylidene difluoride (PVDF) membrane (Invitrogen, United States). The membrane was blocked with 5% non-fat milk blocking buffer and blotted with the following specific antibodies: anti-claudin-1 (Santa Cruz, United States), anti-occludin (Santa Cruz, United States), anti-ZO-1 (Abcam United States), anti-TNF-α (Bio Legend, United States), anti-IFN-γ (Bio Legend, United States), anti-IL-17A (Santa Cruz, United States), anti-TLR4 (Santa Cruz, United States), anti-TRAF6 (Santa Cruz, United States), anti-NF-κB p65 (Santa Cruz, United States), anti-glial fibrillary acidic protein (GFAP, Abcam, United States), anti-vimentin (Abcam, United States), anti-α-SMA (Abcam, United States), anti-TGF-β (Santa Cruz, United States), anti-Col I (Santa Cruz, United States), anti-Col III (Santa Cruz, United States), anti-MMP-2 (Proteintech, United States), anti-TIMP-2 (Santa Cruz, United States), and anti-GAPDH (Santa Cruz, United States) overnight at 4°C. Subsequently, they were incubated with goat anti-rabbit IgG (1:2000)/anti-mouse IgG (1:2000)/anti-rat IgG (1:2000) or mouse anti-goat IgG (1:2000) (Zhongshan Goldenbridge, Beijing, China) for 2 h at room temperature. The protein bands on the membranes were developed using enhanced chemiluminescence detection reagents (Santa Cruz, United States) followed by exposure on Kodak Xdmat blue XB-1 film (Kodak, Rochester, NY, United States). GAPDH was used as a loading control.

### Statistical Analysis

Statistical analyses were performed with SPSS 13.0 software. Data were expressed as mean ± standard deviation (SD). One-way analysis of variance was used to evaluate the differences among groups and two group comparison was determined by Student-Newman-Keuls test. *P* value <0.05 was considered statistically significant.

## Results

### DSS Induced the Intestinal Inflammation in CCl_4_-Induced Liver Fibrosis Mice

**FIGURE 1:**
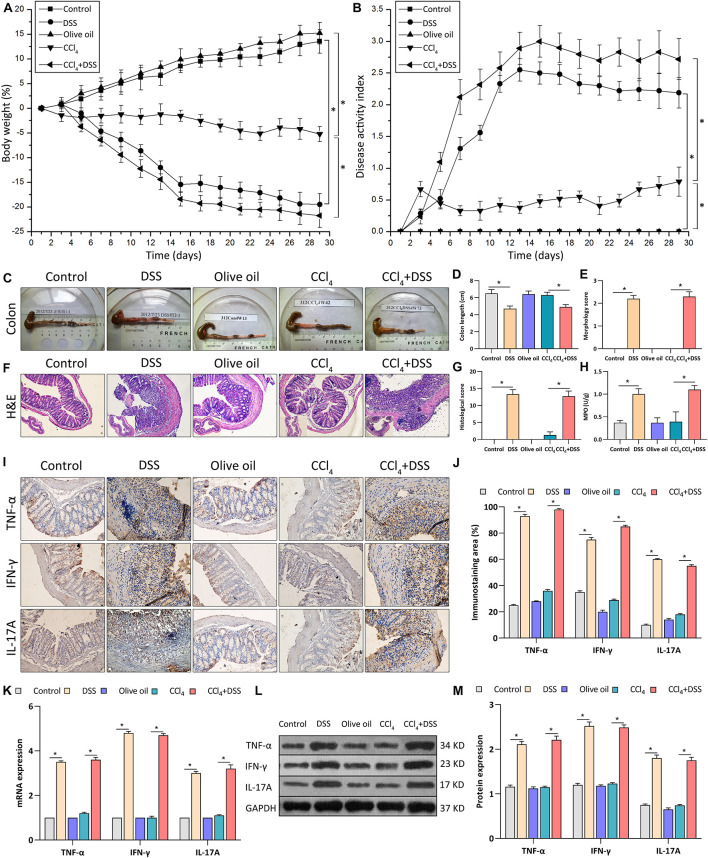
DSS induced the intestinal inflammation in CCl_4_-induced liver fibrosis mice. **(A)** Changes of body weight during the experimental period; **(B)** Disease activity index was evaluated at indicated time points during the experimental period; **(C)** Representative colon specimen of mice from five groups were shown; **(D)** Colon length and **(E)** morphology score were quantified; **(F)** Representative images of H&E staining colon sections (magnifications ×200) and **(G)** histological score were shown; **(H)** Myeloperoxidase activity; **(I)** Representative images of IHC staining colon sections (magnifications ×400) and **(J)** staining intensity of pro-inflammatory cytokines were shown. **(K–M)** Expression levels of pro-inflammatory cytokines in colon were measured by qRT-PCR **(K)** and western blot **(L,M)**. Data were expressed as mean ± SD. *, *p* < 0.05.

To investigate the mechanism of UC in liver fibrosis, liver fibrosis mouse models with intestinal inflammation were established. After that, the severity of intestinal inflammation was evaluated using the indexes including body weight loss, DAI score, histopathological indicators and the expression of proinflammatory cytokines in colon. As shown in Figure 1A, body weights of mice in DSS and CCl_4_ groups decreased significantly compared to mice in their corresponding control group (DSS vs. control, *p* < 0.05; CCl_4_ vs. Olive oil, *p* < 0.05). Notably, we found that DSS treatment significantly aggravated the CCl_4_-induced body weight loss (Figure 1A). Meanwhile, an obvious increase of DAI score were found in DSS and CCl_4_ groups compared to their controls (Figure 1B). Moreover, the increase of DAI after CCl_4_ induction were further enhanced by DSS treatment (Figure 1B).

Through the inspection of colonic specimens, we found that DSS induced the wall thickening, colonic mucosa hyperemia (Figure 1C), decrease of colon length (Figure 1D), and the increase of morphology score (Figure 1E) in CCl_4_-induced liver fibrosis mice. Pathologically, extensive ulceration of the epithelial layer, edema, crypt damage of bowel wall, infiltration of inflammatory cells into the mucosa were observed in DSS and CCl_4_ + DSS group (Figure 1F), accompanied by the higher histological score (Figure 1G). In addition, MPO activities in the colonic tissues of DSS and CCl_4_ + DSS treated mice were significantly increased compared with their control groups (Figure 1H). Furthermore, IHC, qRT-PCR, and western blot analysis all confirmed that the expression levels of proinflammatory cytokines (TNF-α, IFN-γ, and IL-17A) in colon tissues were remarkably increased after DSS treatment (Figures 1I–M). In summary, DSS induced the intestinal inflammation in CCl_4_-induced liver fibrosis mice, implicating the successful establishment of liver fibrosis mouse models with colitis.

### DSS-Induced Colitis Aggravated Liver Damage, Fibrosis and Inflammation in CCl_4_-Induced Liver Fibrosis Mice

**FIGURE 2:**
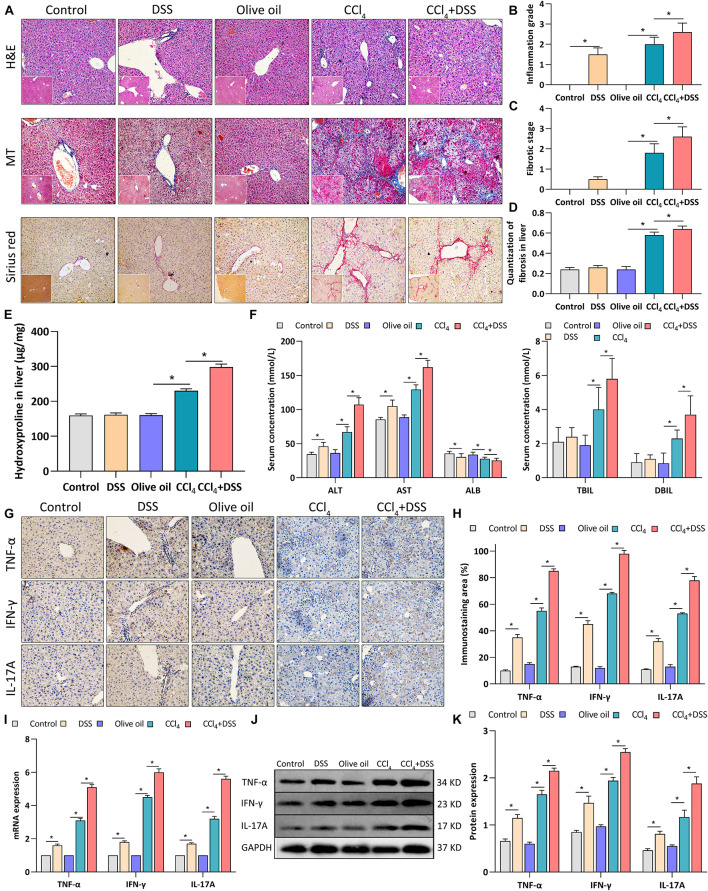
DSS-induced colitis aggravated liver damage, fibrosis and inflammation in CCl4-induced liver fibrosis mice. **(A)** Representative images of H&E staining, Masson’s trichrome (MT) or Sirius red liver sections (magnifications ×200); **(B)** Inflammation grade; **(C)** fibrotic stage and **(D)** quantitative analysis of fibrous tissue were shown; **(E)** The content of hydroxyproline in liver tissue; **(F)** Serum levels of liver function indexes; **(G)** Representative images of IHC staining liver sections (magnifications ×400) and **(H)** staining intensity of pro-inflammatory cytokines were shown. **(I–K)** Expression levels of pro-inflammatory cytokines in liver were measured by qRT-PCR **(I)** and western blot **(J,K)**. Data were expressed as mean ± SD. *, *p* < 0.05.

To assess the effects of DSS-induced colitis on liver inflammation and fibrogenesis in CCl_4_-induced liver fibrosis mice, histopathological changes and expression of proinflammatory cytokines in liver tissues were measured after DSS treatment. Firstly, H&E staining revealed the moderate inflammation in DSS and CCl_4_ group, while the addition of DSS aggravated the inflammation grade in CCl_4_-induced liver fibrosis mice (Figures 2A,B). MT and Sirius red staining showed the more obvious deposition of extracellular matrix (ECM), cell vacuolar degeneration, edema and necrosis around central and portal veins in CCl_4_ + DSS group relative to CCl_4_ group, along with the increases in fibrotic stage and Sirius red positive area (Figures 2A,C,D). Then, tissue hydroxyproline as objective measure of liver fibrosis were detected. As shown in Figure 2E, the addition of DSS further increased the levels of hydroxyproline in CCl_4_-induced liver fibrosis mice. By measuring the liver function indexes, we observed the significant elevation of ALT, AST, TBIL, and DBIL levels, but reduction of ALB in CCl_4_ + DSS group relative to CCl_4_ group, indicating the aggravation of liver damage (Figure 2F). Moreover, expression analysis using IHC, qRT-PCR, and western blot indicated that CCl_4_ + DSS treated mice expressed higher levels of proinflammatory cytokines (TNF-α, IFN-γ, and IL-17A) in liver tissues compared to only CCl_4_ treated mice (Figures 2G–K). Collectively, these results supported the opinion that DSS-induced colitis aggravated liver damage, fibrosis and inflammation in CCl_4_-induced liver fibrosis mice.

### DSS-Induced Colitis Regulated Gut Barrier Function in CCl_4_-Induced Liver Fibrosis Mice

**FIGURE 3:**
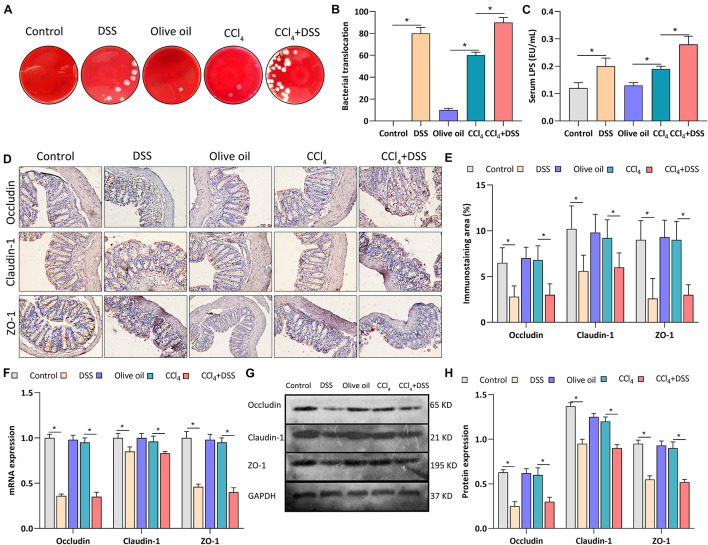
DSS-induced colitis regulated gut barrier function in CCl4-induced liver fibrosis mice. **(A)** Representative images of bacterial translocation assay and **(B)** quantitative analysis of the bacterial translocation to MLN were shown; **(C)** Serum lipopolysaccharide (LPS) levels; **(D)** Representative images of IHC staining colon sections (magnifications ×400) and **(E)** staining intensity of tight junction proteins were shown; **(F–H)** Expression levels of tight junction proteins in colon were measured by qRT-PCR **(F)** and western blot **(G,H)**. Data were expressed as mean ± SD. *, *p* < 0.05.

To assess whether colitis regulated liver fibrosis through gut-liver axis, bacterial translocation, serum LPS level and intestinal epithelial tight junctions were evaluated after DSS treatment. After the bacterial culture of the MLN homogenate, we found that the bacterial translocation to MLN was more frequently present in CCl_4_ + DSS-treated mice compared to CCl_4_ treated mice (Figures 3A,B). We next measured the serum level of LPS, a major recognition marker common to gram-negative bacteria, to determine bacterial translocation. As results, both in DSS and CCl_4_ + DSS groups, serum LPS was significantly higher than that in their controls (Figure 3C). Occludin, claudin-1 and ZO-1 are important tightly linked proteins that play a critical role in maintaining the intestinal epithelial tight junctions and permeability. Therefore, expression levels of these tight junction proteins in colon were detected to evaluate the gut barrier function. Occludin, claudin-1 and ZO-1 staining showed that there were remarkable decrease in the positive staining cells in the livers of DSS and CCl_4_ + DSS treated mice when compared to their controls (Figures 3D,E). Similarly, the identical results were confirmed by qRT-PCR (Figure 3F) and western blot analysis (Figures 3G,H), that CCl_4_ + DSS treated mice had lower expression levels of occludin, claudin-1 and ZO-1 compared to controls. In summary, DSS-induced colitis promoted the bacterial translocation and reduced intestinal epithelial tight junctions, leading to the gut barrier dysfunction in a CCl_4_-induced liver fibrosis mice.

### DSS-Induced Colitis Promoted the Activation of HSCs Through TLR4 Signaling in CCl_4_-Induced Liver Fibrosis Mice

**FIGURE 4:**
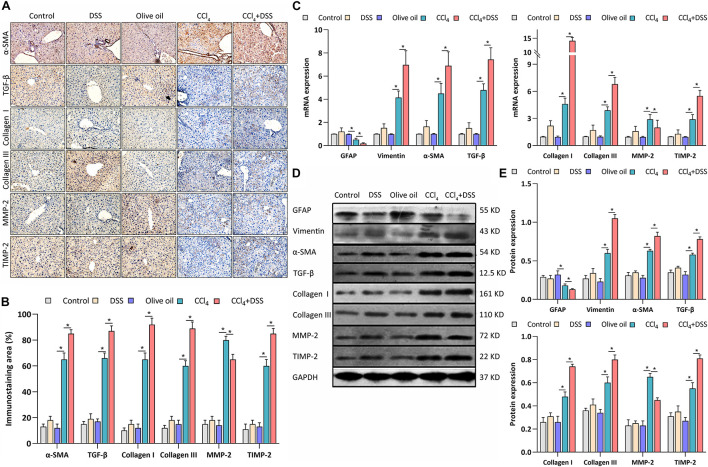
DSS promoted the activation of HSCs in CCl_4_-induced liver fibrosis mice. **(A)** Representative images of IHC staining liver sections (magnifications ×400) and **(B)** staining intensity of proteins-related to HSCs activation were shown; **(C–E)** Expression levels of proteins-related to HSCs activation in liver were measured by qRT-PCR **(C)** and western blot **(D,E)**. Data were expressed as mean ± SD. *, *p* < 0.05.

As previously reported, the expression of α-SMA, GFAP, vimentin, TGF-β1, collagen type I (Col I), collagen type III (Col III), MMP-2, and TIMP-2 are associated with the activation of HSCs. Thus, the expression levels of these proteins were measured after various treatments. From the IHC staining (Figures 4A,B), qRT-PCR (Figure 4C), and western blot (Figures 4D,E), the activation markers, including α-SMA, vimentin, Col I, and Col III, were up-regulated in the livers of CCl_4_-induced liver fibrosis mice than that in the control, which were further enhanced by the DSS exposure. On the contrary, the inactivation marker GFAP was down-regulated in the CCl_4_ group, and was further inhibited in the CCl_4_ + DSS group. In addition, the staining of other HSCs activation related proteins TGF-β1, MMP-2, and TIMP-2 were more pronounced in the livers of CCl4-induced liver fibrosis mice than that in the control. Furthermore, the DSS exposure further increased the CCl_4_-induced up-regulation of TGF-β1 and TIMP-2, while decreased the MMP-2 expression (Figures 4A,B). There results were further confirmed by qRT-PCR (Figure 4C) and western blot (Figures 4D,E).

**FIGURE 5:**
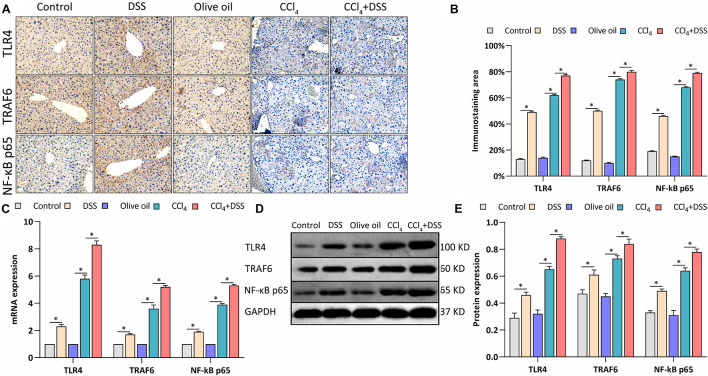
DSS promoted the activation of TLR4 signaling in CCl_4_-induced liver fibrosis mice. **(A)** Representative images of IHC staining liver sections (magnifications ×400) and **(B)** staining intensity of proteins in TLR4 signaling were shown; **(C–E)** Expression levels of proteins in TLR4 signaling in liver were measured by qRT-PCR **(C)** and western blot **(D,E)**. Data were expressed as mean ± SD. ^∗^, *p* < 0.05.

TLR4 signaling was identified in HSCs, and mediates the inflammatory and fibrogenesis. We therefore detected the key proteins related to TLR4 signaling. IHC staining showed a significant enhancement of the TLR4, TRAF6, and NF-κB p65 expression in the livers of mice in DSS group and CCl_4_ group relative to their controls (Figures 5A,B). Meanwhile, the CCl_4_-induced up-regulation of TLR4, TRAF6, and NF-κB p65 were further increased by DSS treatment in the CCl_4_ + DSS group (Figures 5A,B). In addition, similar results were observed from the analysis by qRT-PCR (Figure 5C) and western blot (Figures 5D,E). Given these above results, we speculated that DSS promoted the activation of HSCs by activating the TLR4 signaling pathway in CCl_4_-induced liver fibrosis mice.

## Discussion

**FIGURE 6:**
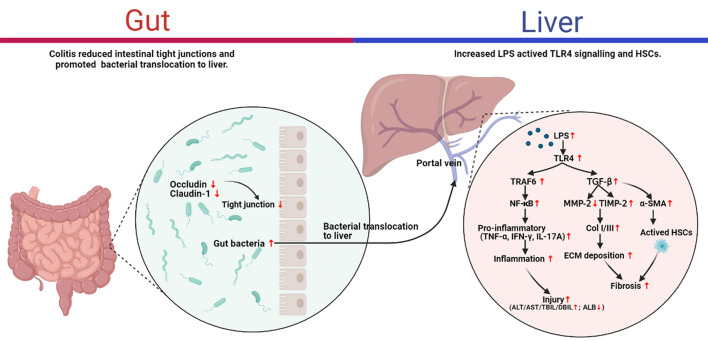
Proposed mechanism for the ulcerative colitis-aggravated liver fibrosis.

Despite there is increasing evidence for a correlation between the gut-liver axis and the pathogenesis of liver diseases, the majority of studies focused on the fibrosis associated with different etiologies, such as ALD, non-alcoholic fatty liver disease (NAFLD), and viral hepatitis (Elpek, 2014). The intuitive illustration of the pathogenesis of liver fibrosis would contribute to the development of anti-fibrotic therapies. To the best of our knowledge, this is the first direct evidence to support the role of DSS-induced colitis in the pathogenesis of liver fibrosis *per se*. The present study highlighted that DSS-induced colitis aggravated the liver fibrosis caused by CCl_4_ through the gut-liver axis. The study of the mechanism indicated that altered intestinal permeability presented as down-regulation of tight junction proteins (occluding, claudin-1) in colitis mice may favor the passage of bacterial products such bacterial LPS into the liver through the portal vein, and then increased LPS activated TLR4 signaling and HSCs activation which involved in liver inflammation and fibrogenesis (Figure 6). These findings provided direct evidence for the role of the gut-liver axis in CCl_4_-induced liver fibrosis.

CCl_4_ is considered to be a direct hepatotoxin that causes central necrosis and steatosis in the liver lobule, ultimately leading to liver fibrosis (Ma et al., 2014). Therefore, in the present study, CCl_4_ was used to establish the experimental liver fibrosis in mice. On the basis of CCl_4_-induced liver fibrosis, we induced chronic colitis by the repeated administration of DSS. As expected, the combination treatment with DSS and CCl_4_ successfully induced intestinal inflammation in mice with liver fibrosis, as evident from the elevated DAI score, bodyweight loss, and enhanced inflammatory response. The intestinal pathology caused by DSS treatment was consistent with the previous reports (Chassaing et al., 2014). Subsequently, various histological and biochemical analyses were conducted on this chronic colitis model with liver fibrosis to verify our assumption.

A considerable number of studies have reported that liver abnormalities including inflammation and fibrosis are typical extra-intestinal manifestations of colitis and are frequently observed (Kummen et al., 2013; Marotto et al., 2020). Here, H&E staining revealed the obvious inflammatory cell infiltration, cytoplasm relaxation, and vacuolar degeneration of hepatocytes in DSS-induced colitis mice, which was confirmed by the enhancement of pro-inflammatory cytokines (TNF-α, IFN-γ, and IL-17A) using IHC, qRT-PCR, and western blot analysis. Therefore, the finding from our study was consistent with the above opinion, that DSS-induced colitis mice presented mild liver inflammation and dysfunction. Serum levels of ALT, AST, and ALB, as markers that assess liver injury (Kasarala and Tillmann, 2016), were also found to be abnormal in DSS-induced colitis mice, further supporting that the liver injury is the extra-intestinal manifestation of colitis. Nevertheless, our results were opposite to the previous studies, which demonstrated that the DSS-induced inflammatory process was mainly restricted to the colon and DSS alone did not induce liver pro-inflammatory cytokines, in turn, affect the liver directly (Karlsson et al., 2008; Gäbele et al., 2011). A possible explanation might be that the high dose and long exposure duration of DSS treatment used in our study caused the immune response, which was completely different from previous studies. In fact, 7-day administration of 3% DSS was identified to induce liver inflammation (Trivedi and Jena, 2013). Additionally, a recent study has also reported that the administration of DSS changed the expression of pro-and anti-inflammatory cytokines such as IL-1β, IL-10, and TGFb-2, suggesting that intestinal inflammation led to liver inflammation (Nii et al., 2020), thus, these results supported our finding again. Besides, we found that DSS alone did not increase the fibrotic stage, Sirius red positive area, and hydroxyproline content compared to control, indicating that DSS alone cannot induce liver fibrosis. These results were consistent with previous studies (Gäbele et al., 2011; Ji et al., 2018); thus, we speculated that DSS alone mainly induced liver injury by mediating the inflammation. More importantly, the liver injury, inflammation, and fibrosis that was originally present in CCl_4_-treated mice were aggravated by the DSS treatment. Actually, this finding was also similar to the data from experimental NASH (Gäbele et al., 2011). The histological abnormalities including ECM deposition, fibrogenesis, inflammatory cell infiltration, and the enhanced inflammatory response caused by colitis were in line with the manifestations in experimental NASH mice (Achiwa et al., 2016). In summary, our study confirmed that DSS-induced colitis aggravated liver damage, fibrosis, and inflammation in CCl_4_-induced liver fibrosis mice.

In the last few years, a growing particular interest has been devoted to the gut-liver axis in liver disease. Gut-liver axis is a complex structure and its alteration seems to play an important role in the progression of liver disease, in particular, of two of its components (gut barrier and microbiota) (Poeta et al., 2017). Theoretically, bacterial translocation is defined as the passage of intestinal bacteria or bacterial products from the gastrointestinal tract to MLN or other extraintestinal sites, which is correlated with the gut barrier function (Porras et al., 2006). In the present study, more intestinal bacteria were found to translocate into MLN in DSS + CCl_4_-treated mice compared to CCl_4_-treated mice, implying that DSS-induced colitis may cause gut barrier dysfunction. Additionally, LPS is a marker for bacterial translocation when detected in the systemic circulation (Alexopoulou et al., 2017). Although LPS in the intestinal cavity is usually unable to penetrate into the healthy intestinal epithelium, it may pass through the intestinal mucosa when the intestinal permeability is disturbed (Stevens et al., 2018). Consistently, the elevated LPS levels in the DSS + CCl_4_-treated mice further supported the bacterial translocation and gut barrier dysfunction. Unfortunately, our present study did not elucidate which type or what bacteria translocate. However, as previously reported, intestinal bacteria that are able to translocate into extraintestinal sites mainly include *Escherichia coli (E. coli), Klebsiella pneumoniae*, and other *Enterobacteriaceae* (Okasha et al., 2000). According to the appearance observation of bacterial colonies, colonial morphology is similar to that of *Enterobacteriaceae*. Analysis of strains using automatic microbial identification systems identified that the stains is mainly *E. coli*. Nevertheless, these attempts are preliminary and the definite type of bacteria translocation remains further investigation.

Moreover, increased intestinal permeability is one of the major mechanisms postulated to promote bacterial translocation (Porras et al., 2006). Meanwhile, tight junctions formed by claudins and occludin play a significant role in the formation and maintenance of intestinal epithelial barrier integrity (Vandenbroucke et al., 2013); thus, the tight junction proteins were measured. Here, occluding, claudin-1 and ZO-1 as main tight junction proteins were obviously down-regulated in the colon after DSS treatment, which further proved the gut barrier dysfunction in the colon. However, it is worth noting that CCl_4_ alone also caused the bacterial translocation and the elevated LPS level, but not the alterations in tight junction proteins in colon. Actually, this finding is not an exception and agrees with the results of other studies (Yao et al., 2006; Fouts et al., 2012). As Fouts et al. reported, occludin was decreased in the small intestine, but not the colon after injection of CCl_4_ as compared to control (Fouts et al., 2012), suggesting that the CCl_4_-induced toxic liver injury could induce the increase of intestinal tight junction permeability, and then promote bacterial translocation. Additionally, Yao et al. have revealed that CCl_4_-treated mice exhibited shortened and thinned microvilli, proposing that the damaged microvillus environment might be also responsible for the early onset of bacterial translocation (Yao et al., 2006). According to the newest evidence, we speculated that the reduction in the thickness of the colonic mucus layer may be another explanation for the bacterial translocation in CCl_4_-treated mice (Haderer et al., 2021). Furthermore, other viewpoints proposed that intestinal bacterial overgrowth and deficiencies in localhost immune defenses are the major mechanisms postulated to favor bacterial translocation in liver disease (Gómez-Hurtado et al., 2011). However, since we mainly focused on the effects of DSS-induced colitis on liver fibrosis, the mechanisms of CCl_4_-induced bacterial translocation were not investigated here. Even so, the present study confirmed that DSS-induced colitis could promote bacterial translocation by inducing the gut barrier dysfunction in colon in CCl_4_-induced liver fibrosis, which is in accord with our hypothesis.

Numerous studies unraveled critical functions for HSCs in the pathogenesis of liver diseases (Khomich et al., 2019). HSCs express neural markers (e.g., GFAP, synemin, and synaptophysin), mesenchymal/mesodermal markers (e.g., desmin vimentin, α-SMA, and Col) (Hyun et al., 2016; Tsuchida and Friedman, 2017). As previously reported, upon activation, HSCs down-regulate neural markers and up-regulate mesenchymal markers, such as vimentin, COl I, and α-SMA (Scholten et al., 2010), being consistent to our present results that GFAP was down-regulated while vimentin, COl I and α-SMA were up-regulated in the DSS + CCl_4_-treated mice. This evidence suggest that the colitis might facilitate the activation of HSCs. Additionally, TGF-β1 is reported to induce the HSC activation (Tsuchida and Friedman, 2017). Here, we observed that DSS induced the further up-regulation of TGF-β1 in the liver fibrosis mice, supporting the activation of HSCs. HSCs are the main cell type that responsible for collagen deposition and fibrogenesis in the liver (Carson et al., 2018). Thus, the increased activation of HSCs accelerated fibrogenesis. Liver fibrosis is the consequence of chronic liver injury and ECM accumulation, such as collagen proteins (Khurana et al., 2021). In the process of liver injury, HSCs are activated to fibroblasts expressing muscle α-SMA under the action of various cytokines and inflammatory mediators, and a large number of ECM components are synthesized during migration and proliferation, mainly Col I and Col III (Gómez-Hurtado et al., 2011; Khurana et al., 2021). The expression of collagen is affected by its related collagenases (MMP-2 and TIMP-2), which regulated collagen fibers (Laronha and Caldeira, 2020). With consistent this mechanism, our study revealed the up-regulation of Col I, Col III, and TIMP-2 and down-regulation of MMP-2 in DSS + CCl_4_-treated mice. Actually, the activation of HSCs is recognized to be mediated by the LPS-activated TLR4 signaling in fibrogenesis (Mann and Marra, 2010). In our study, accompanied by the elevated LPS levels, the up-regulation of TLR4, TRAF6, and NF-κB p65 in TLR4 signaling were also detected, implicating the activation of TLR4/NF-κB signaling. TLR4/NF-κB signaling plays an important role in regulating immune and inflammatory responses (Peng et al., 2014; Khan et al., 2020). Thus, more pro-inflammatory cytokines such as TNF-α, TGF-β, IFN-γ, and IL-17A were released in the liver of DSS + CCl_4_-treated mice. Overall, DSS-induced colitis promoted the activation of HSCs and TLR4 signaling through the gut-liver axis in CCl_4_-induced liver fibrosis mice. These findings were consistent with the data from experimental NASH (Gäbele et al., 2011) and the widely recognized mechanisms (Lee and Friedman, 2011). Thus, the fibrosis associated with different etiologies might share the integrated signaling networks that regulate the ECM deposition. Nevertheless, the investigation of activation of HSCs and signaling mechanism is preliminary, and still remain to be further studied in detail.

## Conclusion

The present study provided the first direct evidence for the pathogenesis of CCl_4_-induced liver fibrosis. We confirmed that intestinal inflammation aggravated liver damage, inflammation, and fibrosis in CCl_4_-induced liver fibrosis mice. More importantly, the results highlighted the role of gut-liver axis in its pathogenesis. Due to the altered intestinal permeability, intestinal inflammation caused by DSS led to the passage of bacterial products into liver, and in turn might promote the activation of TLR4 signaling and HSCs to induce the liver inflammation and fibrogenesis. These direct evidence increased our understanding of liver fibrosis pathogenesis and provided an additional perspective to the development of anti-fibrotic therapies.

## Data Availability Statement

The original contributions presented in the study are included in the article/supplementary material, further inquiries can be directed to the corresponding author.

## Ethics Statement

The animal study was reviewed and approved by The Second Hospital of Hebei Medical University.

## Author Contributions

Y-FL and X-LZ: conceptualization. Y-FL and G-CN: methodology and writing-original draft preparation. Y-FL, C-YL, and J-BG: material preparation. X-LZ: administrative support. G-CN, JS, and HL: formal analysis and data collection. Y-FL, G-CN, and HL: data analysis and interpretation. C-YL and J-BG: writing-review and editing. All authors read and approved the final manuscript.

## Conflict of Interest

The authors declare that the research was conducted in the absence of any commercial or financial relationships that could be construed as a potential conflict of interest.

## Publisher’s Note

All claims expressed in this article are solely those of the authors and do not necessarily represent those of their affiliated organizations, or those of the publisher, the editors and the reviewers. Any product that may be evaluated in this article, or claim that may be made by its manufacturer, is not guaranteed or endorsed by the publisher.
